# The Hydration State of Bone Tissue Affects Contrast in Neutron Tomographic Images

**DOI:** 10.3389/fbioe.2022.911866

**Published:** 2022-06-17

**Authors:** Elin Törnquist, Sophie Le Cann, Alessandro Tengattini, Lukas Helfen, Joeri Kok, Stephen A. Hall, Hanna Isaksson

**Affiliations:** ^1^ Department of Biomedical Engineering, Lund University, Lund, Sweden; ^2^ CNRS, Université Paris Est Créteil, Université Gustave Eiffel, UMR 8208, MSME, F-94010, Créteil, France; ^3^ Institut Laue-Langevin, Grenoble, France; ^4^ Université Grenoble Alpes, CNRS, Grenoble INP 3SR, Grenoble, France; ^5^ Division of Solid Mechanics, Lund University, Lund, Sweden

**Keywords:** neutron tomography, rat tibiae, trabecular bovine bone, image quality, heavy water

## Abstract

Neutron tomography has emerged as a promising imaging technique for specific applications in bone research. Neutrons have a strong interaction with hydrogen, which is abundant in biological tissues, and they can penetrate through dense materials such as metallic implants. However, in addition to long imaging times, two factors have led to challenges in running *in situ* mechanical characterization experiments on bone tissue using neutron tomography: 1) the high water content in specimens reduces the visibility of internal trabecular structures; 2) the mechanical properties of bone are dependent on the hydration state of the tissue, with drying being reported to cause increased stiffness and brittleness. This study investigates the possibility of improving image quality in terms of neutron transmission and contrast between material phases by drying and rehydrating in heavy water. Rat tibiae and trabecular bovine bone plugs were imaged with neutron tomography at different hydration states and mechanical testing of the bone plugs was carried out to assess effects of drying and rehydration on the mechanical properties of bone. From analysis of image histograms, it was found that drying reduced the contrast between bone and soft tissue, but the contrast was restored with rehydration. Contrast-to-noise ratios and line profiles revealed that the contrast between bone tissue and background was reduced with increasing rehydration duration but remained sufficient for identifying internal structures as long as no free liquid was present inside the specimen. The mechanical analysis indicated that the proposed fluid exchange protocol had no adverse effects on the mechanical properties.

## Introduction

Bone is a complex tissue with unique mechanical properties (Rho et al., 1998; van der Linden and Weinans, 2007; Isaksson et al., 2010; Törnquist et al., 2020). Traumas and degenerative disorders reduce bone strength and fracture resistance and often require medical interventions where different types of metallic implants are used (Burr, 2002; Ferretti, 2003; van der Linden and Weinans, 2007). For such interventions to be successful, the structural integrity of the implant fixation is vital (Ryd et al., 1995). The quality of the bone-implant integration can be assessed both by studying the structure of the newly formed peri-implant bone and by evaluating the mechanical properties.

**FIGURE 1:**
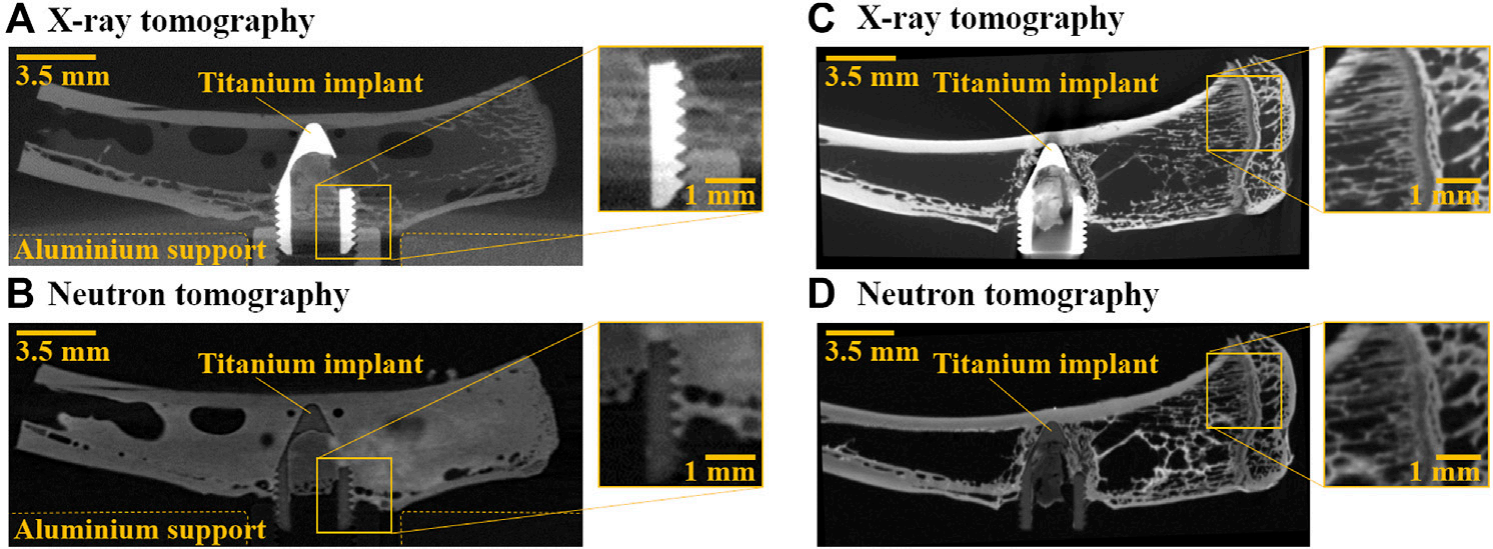
Study motivation. **(A)** X-ray and **(B)** neutron tomography images of a hydrated distal rat tibiae with a titanium implant. In the X-ray image, artifacts close to the metal obscure the bone-implant interface. In the neutron image, no image artifacts occur around the implant, but the free water inside the specimen reduces contrast. **(C)** X-ray and **(D)** neutron tomography images of a dry distal rat tibiae with a titanium implant. As there is no free water inside the specimen, contrast in the neutron image is comparable to that in the X-ray image and allows for visualization of internal structures.

Tomographic imaging is a well-established method for visualizing bone structure, as it is non-destructive and can yield high-resolution 3D images. X-ray tomography is the most used imaging method for bone tissue due to its availability and the contrast it provides between the inorganic mineral in the tissue and the soft tissue and air. However, due to the large difference in X-ray attenuation for tissue and metal, severe image artifacts occur in the vicinity of metallic implants (Figure 1A). Thus, studying the integration of metallic implants into bone using X-rays are challenging (Le Cann et al., 2019). Neutron tomography has recently been shown to be a promising alternative technique to X-ray tomography for the evaluation of bone tissue in close proximity to a metallic implant (Isaksson et al., 2017; Le Cann et al., 2017; Guillaume et al., 2021; Törnquist et al., 2021). Neutrons interact differently with matter compared to X-rays, which translates into an alternative contrast between materials, that is, not correlated with X-ray attenuation values (Bilheux et al., 2009; Roubin et al., 2019; Tengattini et al., 2021). Since most metals used for medical implants (e.g., steel and titanium) are rather transparent when imaged with neutrons, no adverse effects on image quality are seen at the bone-implant interface (Isaksson et al., 2017) (Figure 1B).

The mechanical properties of bone are dependent on the hydration state of the tissue and drying alters the mechanical behavior in terms of increased stiffness and brittleness on the nano-, meso- and macro-scales (Smith and Walmsley, 1959; Broz et al., 1993; Conrad et al., 1993; Nyman et al., 2006; Lievers et al., 2010; Granke et al., 2015; Lin, 2016; Wang et al., 2018). Hence, for physiologically relevant assessment of the mechanical properties, the specimen should be hydrated when tested mechanically. Due to the strong interaction of neutrons with hydrogen, H_2_O appears opaque in neutron tomographic images. This has been utilized, e.g., in studies of fossilized biological specimens (Mays et al., 2017; Zanolli et al., 2017; Urciuoli et al., 2018; Gee et al., 2019; Zanolli et al., 2020). However, because of the inherent hydrogen content in biological tissues, the contrast between tissue and water becomes low (Figure 1B). Furthermore, high hydration levels can cause the specimens to be too opaque to neutrons, which limits transmission and increases noise linked to incoherent scattering. However, as neutrons interact with the atomic nucleus, they are sensitive to isotopic differences (Bilheux et al., 2009). Notably, the interaction between neutrons and hydrogen is stronger than between neutrons and the heavier isotope deuterium. As a result, heavy water (D_2_O) has ∼7 times lower neutron attenuation than normal water (Perfect et al., 2014). This difference in contrast has been exploited, e.g., for tracking water propagation through porous media or plant roots (Tötzke et al., 2019; Tengattini et al., 2021).

We have previously analyzed the quality of bone-implant integration in terms of mechanical stability by performing pull-out tests, both *ex situ* and *in situ* with X-ray and neutron tomography (Le Cann et al., 2017; Le Cann et al., 2019; Raina et al., 2019; Le Cann et al., 2020). To mitigate the aforementioned issues relating to high hydrogen content in bone, whilst retaining physiological hydration of the bone tissue, a protocol was adopted to exchange H_2_O for D_2_O by soaking the specimens for 3 days prior to imaging (Le Cann et al., 2017). The images showed good contrast for bone tissue, but the conclusion was also that D_2_O still reduced contrast between the internal structures where it was overly abundant. However, an alternative explanation may be that the protocol for exchanging H_2_O for D_2_O by soaking was not effective enough, with H_2_O remaining in the specimen and causing reduced contrast.

The aims of the current study were twofold: 1) to elucidate how the amount of D_2_O in bone specimens affects contrast in neutron tomography images, by controlling the amount of H_2_O and D_2_O in the specimens using a drying-rehydrating protocol; 2) to ensure that this sample preparation protocol does not affect the bone mechanical properties.

Visibility of internal structures in neutron tomography images is comparable to X-ray tomography images when the specimens are dried (Figures 1C,D) (Törnquist et al., 2021). Hence, a new exchange protocol was devised in this study where the specimens were first dried to remove as much H_2_O as possible before being rehydrated in D_2_O, thereby improving the exchange compared to the previous diffusion-driven protocol, whilst also allowing for a comparison against the ideal contrast case in images of dried specimens. To this end, distal rat tibiae and trabecular bovine bone plugs were imaged to compare to our previous experimental model (Le Cann et al., 2017; Le Cann et al., 2019; Raina et al., 2019; Le Cann et al., 2020). To our knowledge, few studies have investigated how rehydration after drying affects the mechanical behavior, but the studies that have been performed conclude that rehydration reverts the changes caused by drying (Currey, 1988; Broz et al., 1993; Conrad et al., 1993). To test the effects of the new imaging-optimized rehydration protocol on bone mechanical properties, trabecular bovine bone plugs were subjected to the proposed exchange protocol and subsequently tested in compression. The plugs were extracted from different anatomical locations to obtain varying bone volume fractions (BV/TV) (Hölzer et al., 2012; Bindl et al., 2013; Endo et al., 2016; Manda et al., 2016), as this morphological parameter has a large influence on trabecular bone stiffness (Matsuura et al., 2008; Maquer et al., 2015).

## Materials and Methods

### Samples

**FIGURE 2:**
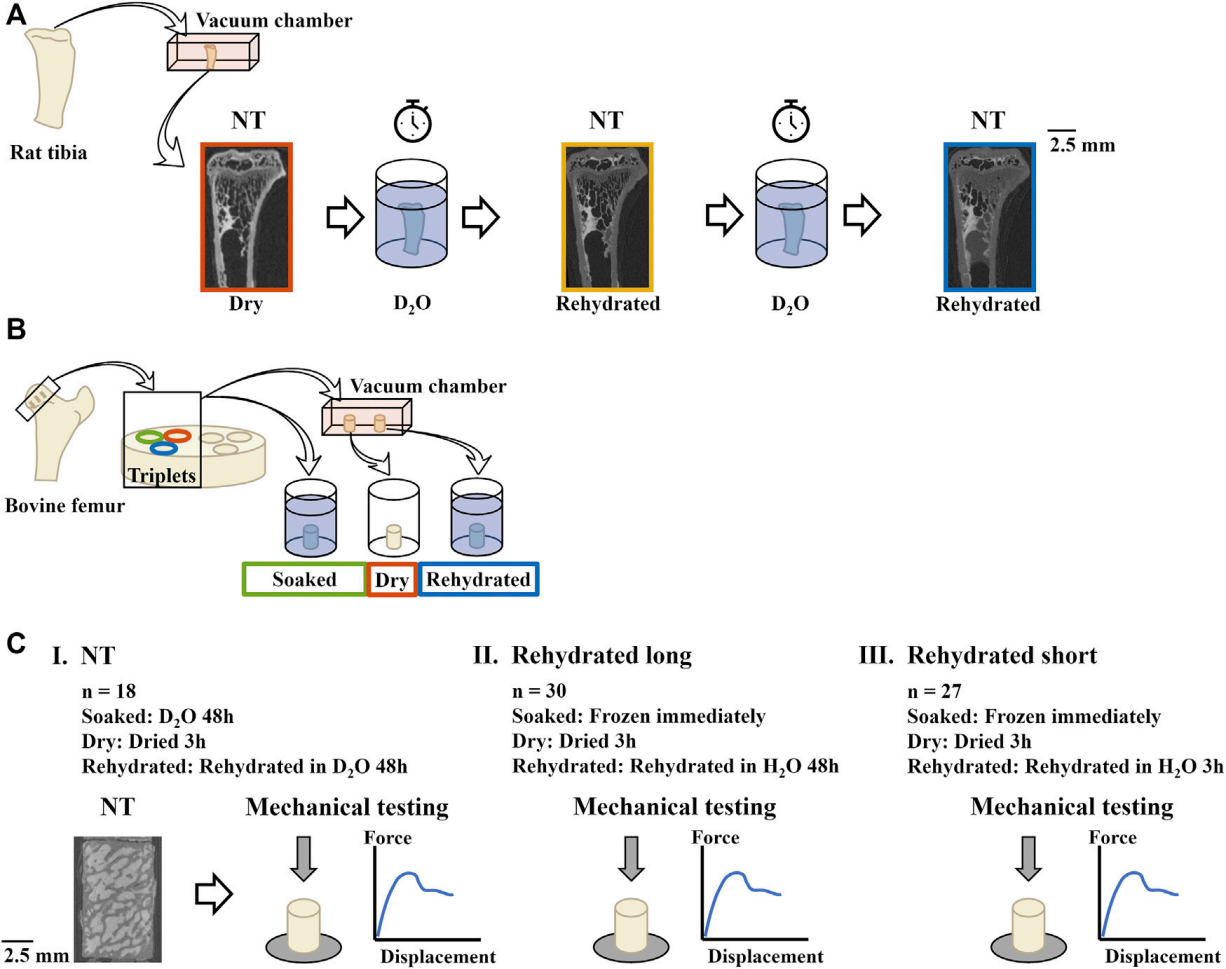
Overview of the experimental procedure. **(A)** Proximal rat tibiae (*n* = 3) were dried in vacuum before being imaged with neutron tomography (NT). The tibiae were then rehydrated in D_2_O and imaged again. Two of the specimens were further hydrated and imaged a third time. **(B)** Trabecular bone plugs (*n* = 75) were extracted from bovine femora. Triplets (plugs extracted next to each other) were divided into three groups which were subjected to three different hydration protocols. **(C)** Overview of the experimental campaigns. **(I)** 18 plugs (*n* = 6 per hydration group) were imaged with NT before being mechanically tested in compression to assess mechanical properties. **(II)** 30 plugs (*n* = 10 per hydration group) were mechanically tested. **(III)** 27 plugs (*n* = 9 per hydration group) were mechanically tested. Note the differences in rehydration duration between **(II)** and **(III)**.

Specimens consisted of proximal tibiae from male Sprague Dawley rats (Figure 2A) and trabecular bovine bone plugs from the proximal femur (Figure 2B).

Sprague Dawley rats (∼10 weeks old, weight 450 ± 39 g) were procured from Taconic (Denmark). The rats were euthanized by CO_2_ asphyxiation. The tibiae (*n* = 3) were dissected, cleaned from soft tissue, and cut approximately 25 mm from the distal epiphysis using a Minitom (Struers). The use of the animals was approved by the Swedish Board of Agriculture (permit numbers: M79-15 and 15288/2019).

The trabecular bovine bone plugs (*n* = 75) were extracted from the major trochanter, head, neck, and metaphysis in the proximal femora of adult (28–29 months) heifers, procured from the local abattoir. The plugs were drilled (RYOBI RDP102L, Techtonic Industries, Germany) using a hollow drill bit (Ø 6 mm) and cut to 10 mm height with a band saw (EXAKT 30/815, Norderstedt, Germany). The plugs were sorted into three groups (one group for each hydration state) to ensure that each group included specimens extracted next to each other (triplets, Figure 2B). Of all the plugs, some (*n* = 18) were imaged with neutron tomography before being mechanically compressed, whereas the rest (*n* = 57) were used only for mechanical testing. The different sets of plugs were tested during three different experimental campaigns (I-III, Figure 2C).

### Hydration Protocol

To explore how the amount of D_2_O in bone specimens affects contrast in neutron tomography images, the rat tibiae were dried in vacuum (−25 inHg) at ambient temperature for 16 h before being imaged with neutron tomography (Figure 2A). After imaging, they were rehydrated by immersion in salinized D_2_O (0.9% NaCl) for ∼12 h and imaged again. Two of the three specimens were rehydrated further and imaged again after ∼30 and ∼34 h, respectively.

To compare the effects of the hydration state on the mechanical properties, the trabecular bone plugs (*n* = 75) were divided into three groups so that three hydration states could be compared: control, dried, and rehydrated (Figure 2B). As no exchange of H_2_O for D_2_O was necessary for the plugs that were not going to be imaged with neutron tomography, the protocols varied slightly, as detailed below.

Experimental campaign I—specimens imaged with neutron tomography (*n* = 18, Figures 2C–I).

• Control (*n* = 6)—soaked in salinized D_2_O for 48 h, to exchange the inherent H_2_O for D_2_O by diffusion.
• Dry (*n* = 6)—dried in vacuum at ambient temperature for 3 h.
• Rehydrated (*n* = 6)—dried in vacuum at ambient temperature for 3 h before being immersed in salinized D_2_O for 48 h.

Experimental campaign II (*n* = 30, Figures 2C–I).

• Control (*n* = 10)—frozen immediately after harvest.
• Dry (*n* = 10)—dried in vacuum (−25 inHg) at ambient temperature for 3 h.
• Rehydrated long (*n* = 10)—dried in vacuum (−25 inHg) at ambient temperature for 3 h before immersion in physiological saline solution (0.9% NaCl, H_2_O) for 48 h.

Experimental campaign III (*n* = 27, Figures 2C–I).

• Control (*n* = 9)—frozen immediately after harvest.
• Dry (*n* = 9)—dried in vacuum (−25 inHg) at ambient temperature for 3 h.
• Rehydrated short (*n* = 9)—dried in vacuum (−25 inHg) at ambient temperature for 3 h before immersion in physiological saline solution (0.9% NaCl, H_2_O) for 3 h.

All specimens were weighed before and after each step of the hydration protocol to assess weight loss and gain due to drying and rehydration (Eq. 1). The change, C, in percent, between the initial dry weight, $w_{\text{i}\text{n}\text{i}\text{t}\text{i}\text{a}\text{l}}$ , and the weight after drying/rehydration, $w_{\text{a}\text{f}\text{t}\text{e}\text{r}}$ , was defined as:

$$\begin{array}{c} C=\frac{w_{\mathrm{after}}-w_{\mathrm{initial}}}{w_{\mathrm{initial}}}\times 100 \end{array}$$ (1)

All specimens were stored frozen (−20°C) until 90 min before being imaged or tested mechanically when they were thawed at room temperature.

### Neutron and X-Ray Tomographic Imaging

Neutron tomography was carried out at the NeXT instrument at Institut Laue-Langevin (ILL) in Grenoble, France (Tengattini et al., 2020). In addition, to obtain bone volume fractions (BV/TV) for the subsequent mechanical analysis, X-ray tomography of all trabecular bovine bone plugs (*n* = 75) was carried out at the NeXT instrument (*n* = 18 plugs) and at the 4D Imaging Lab, Division of Solid Mechanics, Lund University, Sweden (*n* = 57 plugs).

The tibiae (*n* = 3) were imaged individually, wrapped in Teflon tape, and placed inside a sealed Teflon sample holder to mitigate dehydration during imaging. Teflon was used as it is almost transparent to both neutrons and X-rays. Neutron tomography images were acquired using a 10 µm thick Gadolinium scintillator, a pinhole of 30 mm, and a field-of-view (FOV) of 13.5 × 13.5 mm^2^. The collimation distance was ∼10 m, yielding a collimation ratio L/D ≈ 333. 1792 2D projection images were acquired over 360° rotation with an exposure time of 3 s and averaging 3 projections per projection angle to reduce noise. The effective isotropic voxel size was 7.15 µm in the reconstructed 3D images.

The trabecular bone plugs (*n* = 18, 6 per hydration group) were imaged with neutron tomography in groups of three. The specimens were stacked on top of each other and, as for the tibiae, wrapped in Teflon tape and placed inside a sealed Teflon sample holder to mitigate dehydration during imaging. The images were acquired with a pinhole of 23 mm (L/D ≈ 435) and a FOV of 32 × 32 mm^2^. 992 projection images were acquired over 360° rotation with an exposure time of 3.5 s and averaging 3 projections per projection angle to reduce noise. The virtual isotropic voxel size was 15.6 µm in the reconstructed 3D images. The dual neutron and X-ray setup at NeXT enabled corresponding X-ray tomography images to be acquired without moving the specimens. For the X-ray tomography, source voltage and current were 110 kV and 160 μA, respectively. 1312 projections were acquired over 360° rotation, covering the same FOV as the neutron tomography. The frame rate was 9 Hz and 5 projections per angle were averaged to reduce noise. The effective isotropic voxel size was 24 µm in the reconstructed 3D images.

The tomographic reconstructions were performed from the neutron and X-ray radiographs (projections) using a filtered back-projection algorithm with the X-Act software (RX Solutions, Chanavod, France) after normalization for beam inhomogeneities and background noise in the scintillators and cameras. Representative neutron and X-ray images of a specimen from the control group are shown in Supplementary figures 1A,B, respectively.

The trabecular bone plugs (*n* = 57) that were only used for analyzing the mechanical properties (*n* = 19 per hydration group), were imaged with X-ray tomography using a ZEISS Versa XRM520 at the 4D Imaging Lab at Lund University. The specimens were imaged in groups of three, wrapped in gauze (soaked in saline for the control and rehydrated groups) and tape to mitigate changes in hydration during imaging. Source voltage and current were 80 kV and 87 μA, respectively. 1001 projections were acquired over 360° rotation, covering a FOV of 14 × 14 mm^2^, using an exposure time of 1 s. Tomographic reconstruction was performed using a filtered back-projection algorithm with the ZEISS reconstruction software to give 3D image volumes with an isotropic voxel size of 13.5 µm. A representative X-ray image is shown in Supplementary figure 1C.

### Image Analysis

The visibility of internal structures in the neutron tomography images was assessed in terms of contrast between material phases and edge sharpness. Edge sharpness was estimated from line profiles taken in the neutron tomography images of the rat tibiae. Changes in contrast between tissues and background as a function of hydration state in the rat tibiae were evaluated using contrast-to-noise ratio (CNR). Changes in contrast between tissues as a function of hydration state in the trabecular bone plugs were analyzed through the histograms of the images.

Edge sharpness was calculated using line profiles taken from the images of the two tibial specimens that were imaged with neutron tomography three times. To look at similar regions in each sequential scan, the image volumes were manually aligned using ImageJ (1.53c, National Institute of Health, United States). Line profiles (averaging 10 pixels) were taken across a region in each specimen where D_2_O was present in the final hydration state, in a representative image slice identified in all hydration states. The data were smoothed using the Savitzky-Golay method with a span of 9 data points and interpolated using cubic splines. Edges between cortex and background, D_2_O and background, and D_2_O and cortex were identified. The edge spread function (ESF), i.e., the intensity profile across a sharp edge can be assessed through its derivative, the line spread function (LSF). The LSF is bell-shaped and can hence be approximated by a Gaussian distribution which is described by its mean value, µ, and its standard deviation, *σ*. The full-width at half-maximum (FWHM) of the Gaussian then indicates the sharpness of the edge, with a lower value indicating a sharper edge. It can be shown that the FWHM of a Gaussian function can be expressed as

$$\begin{array}{c} FWHM=2\sqrt{2\cdot \ln\left(2\right)}\cdot \sigma \;\approx 2.3548\cdot \sigma \end{array}$$ (2)

Fitting the LSF to raw data can be difficult due to noise, as it is exacerbated by differentiation. However, since a Gaussian function can be used to approximate the LSF, the ESF can be approximated by the integral of the Gaussian function, i.e., the cumulative distribution function (CDF):

$$\begin{array}{c} \Phi \left(x;µ,\sigma \right)=\frac{1}{2}\left(1+erf\left(\frac{x-µ}{\sigma \sqrt{2}}\right)\right) \end{array}$$ (3)

where erf(x) is the error function:

$$\begin{array}{c} erf\left(x\right)=\frac{2}{\sqrt{\pi }}\int_{0}^{x}e^{-t^{2}}\mathrm{dt} \end{array}$$ (4)

Through fitting of Eq. 3 to the intensity profile of the edge, *σ* was obtained and the edge sharpness was assessed through Eq. 2.

CNR was calculated from the reconstructed image volumes of the rat tibiae. For each specimen and hydration state, regions containing only background, cortical bone, or D_2_O were identified in three representative image slices. CNR was calculated for cortex-background, D_2_O-background, and D_2_O-cortex, as

$$\begin{array}{c} CNR=\frac{\left|\mathrm{mean}\left(S_{1}\right)-\mathrm{mean}\left(S_{2}\right)\right|}{\sqrt{\mathrm{sd}{\left(S_{1}\right)}^{2}+\mathrm{sd}{\left(S_{2}\right)}^{2}}} \end{array}$$ (5)

where $S_{i=1,2}$ are the grey value distributions of the contrasting material phases.

The neutron tomography images of the bovine trabecular bone plugs were analyzed in terms of grey value distributions by comparing the histograms (250 bins) of the images, excluding the background.

The bone volume fractions (BV/TV) of the bone plugs were calculated from the X-ray tomography images using the ImageJ plugin BoneJ (v7.09). The images were filtered using a median filter with a radius of 2 pixels, after which the bone was segmented in the images using thresholding. For the bone plugs imaged at NeXT-Grenoble, the grey value interval for bone was set to 30375-65535 (16-bit images), and for those imaged at the 4D Imaging Lab, it was set to 53500-65535.

### Mechanical Testing

All trabecular bone plugs were tested in unconfined uniaxial compression using a 20 kN load cell (Instron® 8511.20, Instron Corp). After applying a pre-load of 15 N, a constant displacement rate of 1 mm/s was used to compress the specimens. From the collected force-displacement data, intrinsic parameters were calculated by normalization to specimen dimensions. Failure was considered as the first clear peak in the stress-strain curves. Elastic modulus was calculated from the slope of the linear portion of the stress-strain curve. Toughness was calculated as the area under the curve until failure. Only compression tests where the load curve showed a clear peak was included in analysis. This resulted in omitting four specimens in the soaked group, two in the rehydrated group, and four in the dried group. Mechanical testing of the plugs imaged with neutron tomography (*n* = 6 per hydration group), the plugs with rehydration duration 48 h (*n* = 10 per hydration group), and the plugs with rehydration duration 3 h (*n* = 9 per hydration group) was done at three different occasions (Figure 2C).

### Statistical Analysis

The mechanical properties were compared between hydration groups through a linear mixed-effects model in Matlab (R2019a, MathWorks Inc., MA, United States). The mechanical parameter was defined as the response/dependent variable. BV/TV and hydration state were considered explanatory variables and experimental campaign was used as a random effect to account for possible differences between campaigns. The response variable was log-transformed to correct for heteroskedasticity in the residuals, after which assumptions of homoskedasticity and normality were met (Supplementary figures 2A,B). A quadratic effect of BV/TV was added to correct for quadratic pattern to the response-versus-BV/TV plots. Goodness-of-fit for the linear mixed-effects model was assessed based on root-mean-square-error (RMSE), and adjusted coefficient of determination (adjusted R^2^). Goodness-of-fit for the edge sharpness analysis was assessed based on normalized root-mean-square-error (NRMSE). All statistics were assessed at a significance level of *p* = 0.05.

## Results

The neutron tomography images of the rat tibiae showed a decrease in contrast between bone tissue and other material phases with increasing hydration. The neutron tomography images of the trabecular bovine bone plugs showed a decrease in contrast between bone tissue and marrow in the dry specimens. Rehydration reverted the contrast back to the level observed in the soaked specimens. The mechanical analysis showed no significant differences between hydration states.

### Assessment of Image Quality

**FIGURE 3:**
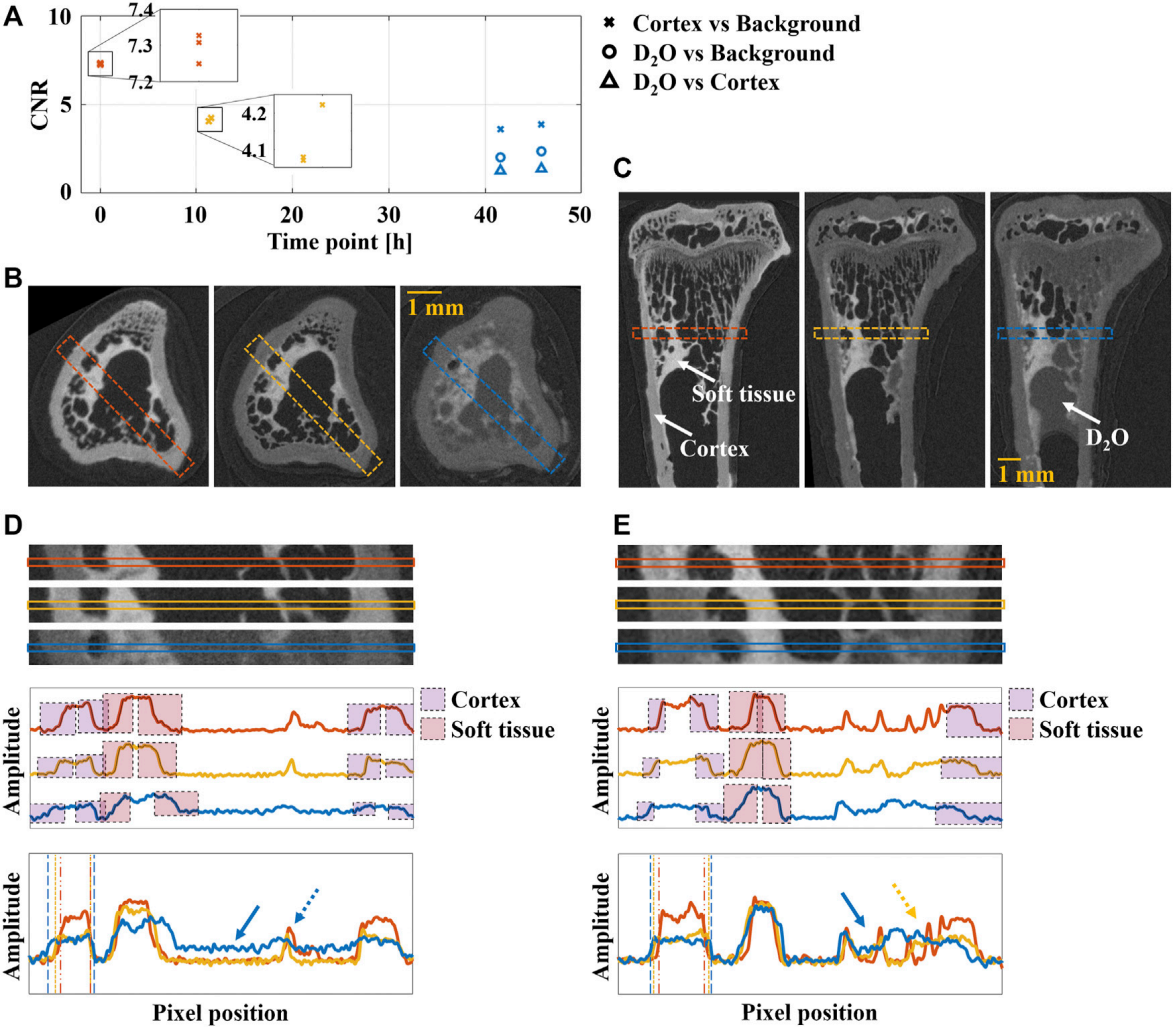
Image analysis of the rat tibiae at different hydration states: dry—red, rehydrated 12 h—orange, rehydrated > 40 h—blue. **(A)** CNR for cortex vs. background, D_2_O vs. background, and D_2_O vs. cortex at the different hydration states, with zoom-ins of the first two hydration state for easier identification of the individual data points. Free D_2_O was only noticed in the third set of images of the two tibiae that were rehydrated for > 40 h (shown in B and C). **(B)** Transverse and **(C)** sagittal slices of tibia with regions for line profiles indicated with dashed square. Cortex, soft tissue, and D_2_O are indicated with white arrows. **(D)** and **(E)** Zoom-in of the regions in B and C, respectively, with corresponding separated and overlaid line profiles. Edges used for edge sharpness analysis are indicated with colored squares (cortex—purple, soft tissue—red). In the overlaid line profiles, thickening of the cortex with increased hydration is indicated with dashed lines. The arrows indicate increased signal due to D_2_O (solid), and reduced contrast for inner structures (dashed). The yellow dashed arrow indicates reduced contrast for inner structures after 12 h of rehydration.

CNR analysis of the neutron tomography images of the rat tibiae showed a decrease in contrast between the cortex and the background with increasing hydration of the specimens (Figure 3A). After rehydration for > 40 h, free D_2_O was seen in the intramedullary canal (Figures 3B,C). At this time point, CNR between cortex and D_2_O, and between background and D_2_O, were lower than between cortex and background.

The line profile analysis of the neutron tomography images of the rat tibiae showed decreased contrast between bone tissue and background with increasing rehydration (Figures 3B–E). When the intramedullary canal was filled with D_2_O, contrast was only seen between D_2_O and soft tissue, due the increased signal from the D_2_O compared to the background (Figures 3D,E). In addition, thickening of the cortex as the tissue was rehydrated was observed. Edge sharpness appeared to be similar for all hydration states (Table 1).

**TABLE 1:** Edge sharpness analysis. FWHM for the edges indicated in Specimen I (Figure 3D) and Specimen II (Figure 3E). RC—right cortex (purple in Figures 3D,E), ST—soft tissue (red in Figures 3D,E), LC—left cortex (purple in Figures 3D,E). A lower FWHM indicates a sharper edge.

	RC		ST		LC		NRMSE
	Left	Right	Left	Right	Left	Right	
Specimen I							
Dry	0.72	0.70	0.70	0.69	0.74	0.71	0.07 ± 0.005
Rehydrated 12 h	0.74	0.74	0.68	0.68	0.75	0.63	0.08 ± 0.02
Rehydrated 42 h	0.78	0.66	0.75	0.75	0.70	0.75	0.16 ± 0.17
Specimen II							
Dry	0.69	0.73	0.69	0.60	-	0.73	0.10 ± 0.07
Rehydrated 12 h	0.72	0.73	0.72	0.68	-	0.77	0.08 ± 0.02
Rehydrated 47 h	0.75	0.75	0.74	0.71	-	0.79	0.08 ± 0.006

**FIGURE 4:**
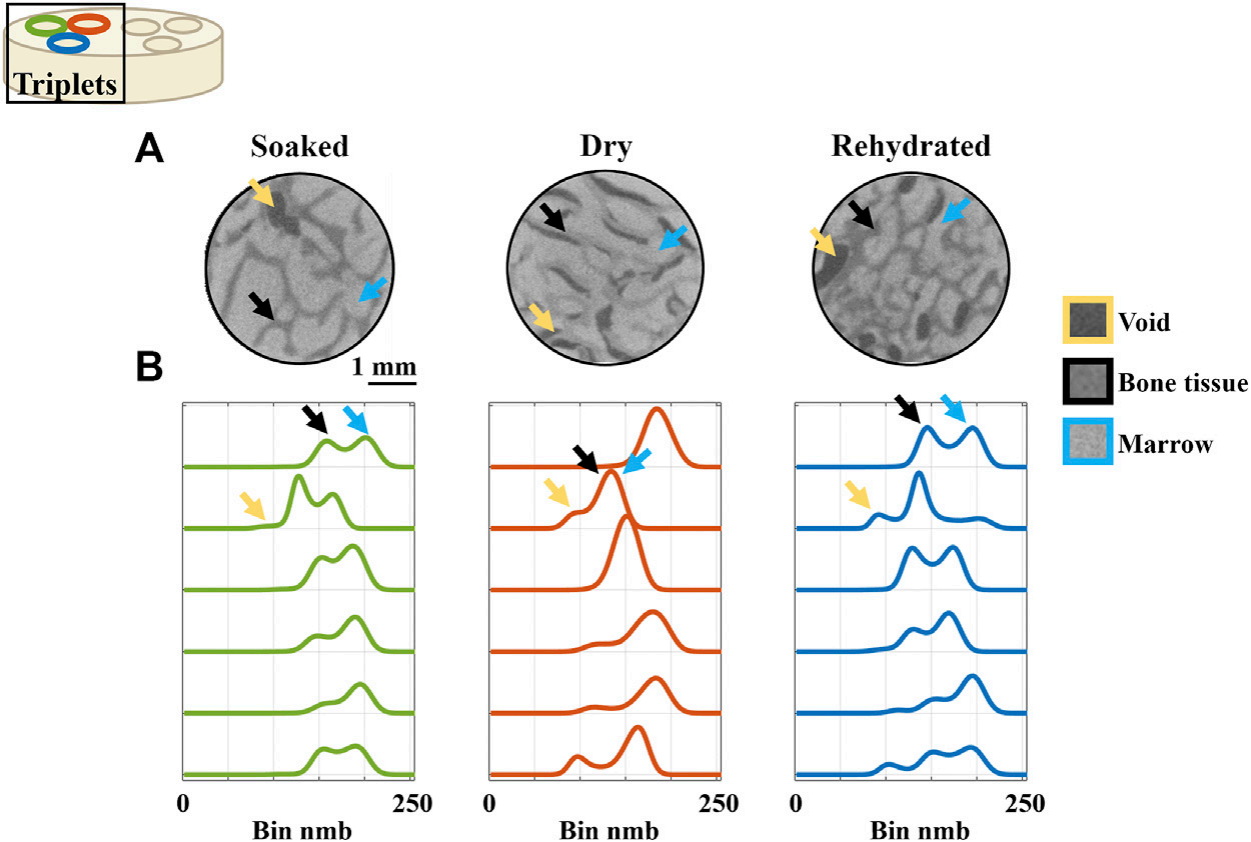
Histograms of the neutron tomography images of the trabecular bovine bone plugs. **(A)** Transverse slices of representative specimen from each hydration group. **(B)** Specimen-specific histograms. Each line corresponds to one specimen (*n* = 6 per hydration group). Each row corresponds to one triplet, i.e., plugs extracted next to each other from the same anatomical location. Arrows indicate peaks corresponding to void (yellow), bone tissue (black), and marrow (blue).

The images of the trabecular bovine bone plugs (Figure 4A) showed decreased contrast between bone tissue and marrow in the dry group compared to the soaked group (control). Rehydration reverted the contrast to similar levels as the soaked group. The histograms of the trabecular bone plugs displayed peaks corresponding to void, bone tissue, and marrow (Figure 4B). The soaked specimens lacked the peak corresponding to void, except in one out of six specimens where a vague peak was observed; the void was identified in the corresponding image volume and is indicated in Figure 4A. The dried specimens had more prominent void peaks and displayed a merging of the peaks corresponding to bone tissue and marrow. The rehydrated specimens exhibited more noticeable void peaks than the soaked group with well separated bone tissue and marrow peaks. The differences in void fractions were evident also from visual inspection of the images, where numerous voids could be seen in the specimens from the dry and rehydrated groups as opposed to the soaked group (Figure 4A).

### Mechanical Properties

**FIGURE 5:**
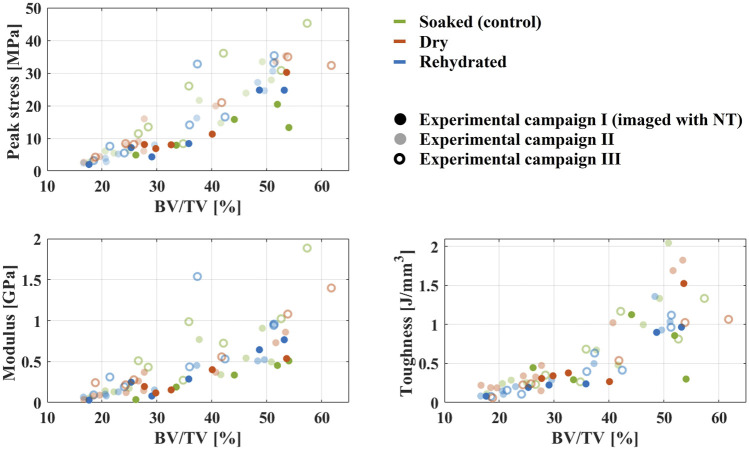
Mechanical properties as a function of bone volume fraction (BV/TV). Hydration groups are color coded as soaked—green, dried—red, rehydrated—blue. The specimens imaged with neutron tomography (tested during experimental campaign I) are shown in opaque colors. The specimens tested during experimental campaign II are shown in transparent colors. The specimens tested during experimental campaign III are shown as outlined circles.

For assessment of mechanical properties, the peak stress, Young’s modulus, and toughness were compared between hydration groups (Figure 5). Statistical analysis showed no differences between the hydration states. BV/TV and (BV/TV)^2^ were significant determinants for all parameters (*p* < 0.005). Moreover, the analysis indicated that there was an effect from the different experimental campaigns (*p* < 0.002). Each parameter’s model had high goodness-of-fit (Table 2). Comparisons between response and fitted values can be found as Supplementary figure 2C.

**TABLE 2:** Goodness-of-fit of the mixed effects models used to analyze the mechanical parameters.

Peak stress	Young’s modulus	Toughness
Adjusted R^2^ = 0.91 RMSE = 0.25	Adjusted R^2^ = 0.83 RMSE = 0.39	Adjusted R^2^ = 0.83 RMSE = 0.36

## Discussion

Proximal rat tibiae and trabecular bovine bone plugs were imaged with neutron tomography at different states of hydration with the aim of elucidating the effects of hydration state on the contrast between the material phases. The trabecular bone plugs were subsequently subjected to compression testing to investigate possible changes with respect to their mechanical properties.

### Image Quality

For the tibiae samples, CNR showed that the contrast between bone tissue and background decreased with increasing rehydration (Figure 3). Clear amplitude differences in the line profile analysis indicated that the CNR after > 40 h of rehydration was high enough to differentiate between background and bone tissue. However, in regions where free liquid was present in the intramedullary canal, CNR between liquid and bone tissue was too low to allow for differentiation between the two. This indicates a continued rehydration of the tissue after the initial 12 h of rehydration. Indeed, at this time point the weights of the tibiae were 10.4 ± 1.8% (mean ± std) less than before drying. Furthermore, it shows that contrast between bone tissue and background is high enough for identification of internal structures, as long as no free liquid is present inside the specimen. It is important to note that the method used to remove H_2_O, i.e., drying in vacuum at room temperature, only partially liberates bound water (Nyman et al., 2013). During the rehydration, such bound H-atoms are released into the rehydrating liquid. Hence, rehydration using D_2_O following drying at room temperature results in a liquid containing both H and D. This method of dehydration was chosen because of its less intrusive nature to minimize influence of the mechanical properties of the tissue. Further studies are needed to investigate the effects of removing more of the bound water on neutron image quality and to assess its impact on the mechanical properties of the tissue. Water content, distribution, and D/H ratio can be deduced directly from the grayscale variations in both neutron and x-ray tomography images but require knowledge of the chemical composition of the sample as well as careful calibration of the images (Defraeye et al., 2014; Oesch et al., 2019). Given the complex chemical composition, in combination with biological variation, of the bone samples in the current study, assumptions about compositional heterogeneity and distribution would be necessary. Hence, the accuracy of such measurements would be low compared to that in (Defraeye et al., 2014) where a simpler system was analyzed. Furthermore, the calibration method described in (Oesch et al., 2019) requires reference objects that are not affected by moisture change to be present in the specimens, and would need further development due to the swelling of the tissue. Still, the methods adopted in the aforementioned papers present interesting stepping stones. Nevertheless, our findings show that removal of free liquid inside the specimen enables successful image analysis, regardless of the hydrogen isotope. The soft tissue remained clearly differentiable from both background and liquid for all hydration states, supposedly due to a comparatively high hydrogen content.

For the trabecular bone plugs, the histograms showed three main peaks, corresponding to internal voids, bone tissue, and marrow (Figure 4). The contrast between bone tissue and marrow decreased with drying of the trabecular bone plugs. This was evident both from visual inspection and attempts at segmentation of the tissues by grey value thresholding. Coherently, the histograms for the dry specimens showed merging of the peaks corresponding to bone tissue and marrow (black and blue arrows in Figure 4B). More interestingly, no apparent differences could be seen between the soaked (control) and rehydrated groups. This indicates that exchange of H_2_O for D_2_O may be equally efficient by diffusion as by drying and rehydration. The peak corresponding to internal voids (blue arrows in Figure 4B) was absent or of very low frequency for the specimens in the control group. Due to the presence of bone marrow, little internal voids were expected in this group. With drying, more internal voids were present than in the control group as marrow was found to shrink and detach from the trabeculae. After rehydration, internal voids were diminished but still present to a higher degree than in the control group. This was seen both by visual inspection and in the histograms for the rehydrated specimens, which displayed more prominent peaks corresponding to internal voids.

### Mechanical Properties

The mechanical properties were expected to be affected by the hydration state, as dried bone is expected to become stiffer and more brittle (Smith and Walmsley, 1959; Broz et al., 1993; Conrad et al., 1993; Nyman et al., 2006; Lievers et al., 2010; Granke et al., 2015; Lin, 2016; Wang et al., 2018). Conrad et al. (1993) assessed changes in mechanical behavior of trabecular bone due to freeze-drying and found that freeze-drying followed by rehydration for 24 h caused a 24% increase in stiffness compared to freshly frozen controls and that non-rehydrated freeze-dried specimens were 33% stiffer than rehydrated specimens (Conrad et al., 1993). Smith et al. (1959) deduced a 7% increase in stiffness during air-drying of cortical bone for 5 h (Smith and Walmsley, 1959). In the current study, no effect on the mechanical properties as a function of hydration state were seen. The freeze-drying process used in Conrad et al. (1993) took 6 days and supposedly led to a larger decrease in moisture compared to the 3 h of drying in vacuum in the current study. However, the 5 h of drying in air used by Smith et al. (1959) should have caused a similar loss of moisture as in the current study. Hence, it would appear as if the degree of drying does not explain the lack of differences seen here. Still, the influence of bone marrow on the mechanical properties of the trabecular bone plugs could possibly account for the discrepancy compared to Smith et al. (1959), as their tests were performed on cortical bone. Hence, our results indicate that drying for 3 h does not substantially affect the mechanical properties of the type of specimens in our study.

**FIGURE 6:**
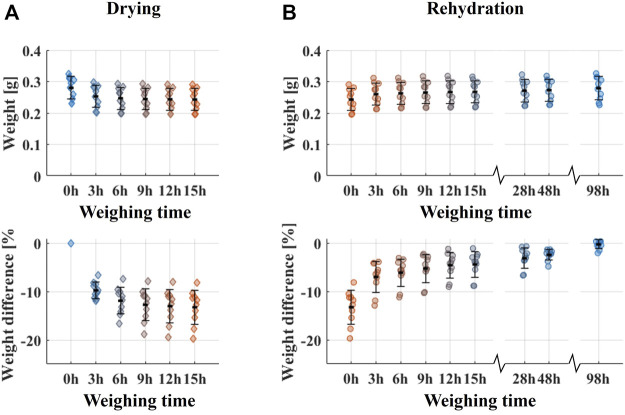
Weight changes due to drying and rehydration of 10 extra trabecular bovine bone plugs. **(A)** Weight and weight difference compared to original weight during drying. **(B)** Weight and weight difference compared to original weight (before drying) during rehydration.

There was a noticeable effect of drying on the neutron images, with reduced contrast between bone and marrow, as compared to the soaked and rehydrated groups (Figure 4). This shows that drying for 3 h yields adequate water extraction for there to be an impact on how the neutrons interact with the tissue. During the drying process, the trabecular bone plugs lost 10.0 ± 2.9% (mean ± std) in weight. Drying of an additional 10 trabecular bone plugs for 15 h and weighing them every 3 h showed that most water content was indeed lost during the first 3 h, with weights of the specimens decreasing by 9.7 ± 1.7% in that timeframe (Figure 6A).

Upon drying, marrow was found to detach from the trabeculae, resulting in more internal voids than in the control group. This was seen both by visual inspection of the tomographic images and from the histogram analysis. A prominent peak corresponding to the grey values in the voids was seen in the histograms of the specimens from the dried group but was absent in the histograms of the soaked specimens. These changes were not fully reversed after rehydration, as seen both in the tomographic images and in the histograms where the void peak was more prominent in the rehydrated group than in the soaked group. Hence, rehydration did not result in full reversion of structural changes due to drying. However, these changes in marrow distribution appear not to have influenced the mechanical properties as, again, no differences were seen between the hydration states.

The trabecular bone plug specimens were found to weigh 4.3 ± 6.4% less after rehydration compared to the original state. The durations of the rehydration (3 and 48 h) were chosen to match our previous exchange protocol (Le Cann et al., 2017) as full rehydration was expected to have occurred after 3 h (Broz et al., 1993). Consequently, any changes to the mechanical properties caused by the drying were expected to have been reverted at the time of mechanical testing. Rehydration of the 10 additional bone plugs used to evaluate the weight loss during drying showed that 48.9 ± 9.9% of the lost weight was regained after 3 h and 81.9% ± 5.3% after 48 h (Figure 6B). After 98 h, the difference in weight compared to the initial state was only 0.2 ± 0.95%. This showed that almost all weight changes were reverted by continued rehydration. However, as no differences in mechanical properties were observed between the soaked and rehydrated groups, prolonging the rehydration duration appeared unnecessary.

The mixed-effects models showed that BV/TV was a significant predictor for all investigated mechanical parameters. Indeed, BV/TV is known to have a large influence on the mechanical properties of trabecular bone (Matsuura et al., 2008; Maquer et al., 2015). The models also showed a quadratic dependence on BV/TV, indicating that its influence varies depending on its value. As an example, a lower BV/TV equates to more marrow being present in the specimen. As both BV/TV and marrow influence the mechanical properties (Halgrin et al., 2012), different ratios of the two would have different effects on the compression response of the specimens. Furthermore, the statistical analysis showed that there were differences in mechanical properties between experimental campaigns. As trabecular ultrastructure is optimized for the loading conditions occurring in the specific anatomical site, the mechanical properties vary depending on anatomical origin (Morgan et al., 2003; Endo et al., 2016). However, all bone plugs were extracted from the same anatomical location. Nevertheless, it is possible that some of the differences between experimental campaigns could be attributed to inter-animal differences as the femora used to obtain the bone plugs came from different animals and each campaign used a different femur.

### Limitations

Due to time constraints, the neutron tomography images of the specimen rehydration for 42 h were acquired with a slightly reduced number of projections (1408 compared to 1792). However, this was still enough to fulfill the Shannon theorem (Bilheux et al., 2009). In addition, the noise levels (estimated from a region containing only background, as the standard deviation of the grey value distribution) were similar for all specimens and hydration states (2250.6 ± 91.0, mean ± std). Consequently, we are confident that the image quality was not reduced significantly. Furthermore, due to movement during the acquisition of the second set of dry trabecular bone plug specimens, the images were reconstructed using 30% fewer projections, resulting in slightly noisier data. However, as for the tibiae, noise levels were similar for all specimens and hydration states (1192.4 ± 93.2). For the mechanical analysis, only BV/TV was used as a determining factor. Albeit being a morphological parameter known to have a major impact on the mechanical properties of trabecular bone, other structural parameters, such as, e.g., anisotropy and trabecular thickness, also influence the mechanical behavior of these types of specimens (Matsuura et al., 2008; Maquer et al., 2015). A larger sample set than what was used in this study would allow for a more complete characterization of structural parameters.

## Conclusion

This study investigated how the amount of D_2_O in bone specimens affects contrast in neutron tomography images and assessed if the method used to exchange H_2_O for D_2_O, i.e., drying, alters the bone mechanical properties. Neutron tomography images were acquired of bone specimens at different hydration states and the visibility of internal structures was assessed. The findings showed that exchanging H_2_O for D_2_O does not improve visibility of internal structures in these bone specimens and that the contrast between free D_2_O and trabecular bone is insufficient for structural characterization. Instead, the analysis showed that presence of free water inside the specimen causes reduced contrast between bone tissue and surrounding structures. Hence, we conclude that it is vital to reduce the amount of free water inside the specimens, regardless of hydrogen isotope. Furthermore, the mechanical analysis showed that the short duration (3 h) of drying used in the study did not affect the mechanical properties of these bone specimens. However, additional tests on different samples are necessary to corroborate that this is conclusion holds not only for the specimen types considered in this study.

## Data Availability

The datasets presented in this study can be found in the Institut Laue-Langevin online repository, doi:10.5291/ILL-DATA.UGA-100.

## References

[B1] Bilheux H. Z. McGreevy R. Anderson I. S. (Editors) (2009). Neutron Imaging and Applications - A Reference for the Imaging Community (Berlin, Germany: Springer US). 10.1007/978-0-387-78693-3

[B2] Bindl R. Oheim R. Pogoda P. Beil F. T. Gruchenberg K. Reitmaier S. (2013). Metaphyseal Fracture Healing in a Sheep Model of Low Turnover Osteoporosis Induced by Hypothalamic-Pituitary Disconnection (HPD). J. Orthop. Res. 31, 1851–1857. 10.1002/jor.22416 23813786

[B3] Broz J. J. Simske S. J. Greenberg A. R. Luttges M. W. (1993). Effects of Rehydration State on the Flexural Properties of Whole Mouse Long Bones. J. Biomech. Eng. 115, 447–449. 10.1115/1.2895510 8309241

[B4] Burr D. B. (2002). Bone Material Properties and Mineral Matrix Contributions to Fracture Risk or Age in Women and Men. J. Musculoskelet. Neuronal Interact. 2, 201–204. 15758433

[B5] Conrad E. U. Ericksen D. P. Tencer A. F. Strong D. M. Mackenzie A. P. (1993). The Effects of Freeze-Drying and Rehydration on Cancellous Bone. Clin. Orthop. Relat. Res. 290, 279–284. 10.1097/00003086-199305000-00036 8472461

[B6] Currey J. D. (1988). The Effects of Drying and Re-wetting on Some Mechanical Properties of Cortical Bone. J. Biomechanics 21, 439–441. 10.1016/0021-9290(88)90150-9 3417696

[B7] Defraeye T. Derome D. Aregawi W. Cantré D. Hartmann S. Lehmann E. (2014). Quantitative Neutron Imaging of Water Distribution, Venation Network and Sap Flow in Leaves. Planta 240, 423–436. 10.1007/s00425-014-2093-3 24923675

[B8] Endo K. Yamada S. Todoh M. Takahata M. Iwasaki N. Tadano S. (2016). Structural Strength of Cancellous Specimens from Bovine Femur under Cyclic Compression. PeerJ 4, e1562. 10.7717/peerj.1562 26855856 PMC4741075

[B9] Ferretti J. (2003). Bone Mass, Bone Strength, Muscle-Bone Interactions, Osteopenias and Osteoporoses. Mech. Ageing Dev. 124, 269–279. 10.1016/S0047-6374(02)00194-X 12663124

[B10] Gee B. M. Bevitt J. J. Garbe U. Reisz R. R. (2019). New Material of the 'microsaur' Llistrofus from the Cave Deposits of Richards Spur, Oklahoma and the Paleoecology of the Hapsidopareiidae. PeerJ 7, e6327–51. 10.7717/peerj.6327 30701139 PMC6348957

[B11] Granke M. Does M. D. Nyman J. S. (2015). The Role of Water Compartments in the Material Properties of Cortical Bone. Calcif. Tissue Int. 97, 292–307. 10.1007/s00223-015-9977-5 25783011 PMC4526331

[B12] Guillaume F. Le Cann S. Tengattini A. Törnquist E. Falentin-Daudre C. Albini Lomami H. (2021). Neutron Microtomography to Investigate the Bone-Implant Interface-Comparison with Histological Analysis. Phys. Med. Biol. 66, 105006. 10.1088/1361-6560/abf603 33831846

[B13] Halgrin J. Chaari F. Markiewicz É. (2012). On the Effect of Marrow in the Mechanical Behavior and Crush Response of Trabecular Bone. J. Mech. Behav. Biomed. Mater. 5, 231–237. 10.1016/j.jmbbm.2011.09.003 22100098

[B14] Hölzer A. Pietschmann M. F. Rösl C. Hentschel M. Betz O. Matsuura M. (2012). The Interrelation of Trabecular Microstructural Parameters of the Greater Tubercle Measured for Different Species. J. Orthop. Res. 30, 429–434. 10.1002/jor.21525 21834128

[B15] Isaksson H. Harjula T. Koistinen A. Iivarinen J. Seppänen K. Arokoski J. P. A. (2010). Collagen and Mineral Deposition in Rabbit Cortical Bone during Maturation and Growth: Effects on Tissue Properties. J. Orthop. Res. 28, 1626–1633. 10.1002/jor.21186 20540098

[B16] Isaksson H. Le Cann S. Perdikouri C. Turunen M. J. Kaestner A. Tägil M. (2017). Neutron Tomographic Imaging of Bone-Implant Interface: Comparison with X-Ray Tomography. Bone 103, 295–301. 10.1016/j.bone.2017.07.022 28739417

[B17] Le Cann S. Tudisco E. Perdikouri C. Belfrage O. Kaestner A. Hall S. (2017). Characterization of the Bone-Metal Implant Interface by Digital Volume Correlation of *In-Situ* Loading Using Neutron Tomography. J. Mech. Behav. Biomed. Mater. 75, 271–278. 10.1016/j.jmbbm.2017.07.001 28759839

[B18] Le Cann S. Tudisco E. Tägil M. Hall S. A. Isaksson H. (2020). Bone Damage Evolution Around Integrated Metal Screws Using X-Ray Tomography - *In Situ* Pullout and Digital Volume Correlation. Front. Bioeng. Biotechnol. 8, 1–10. 10.3389/fbioe.2020.00934 32850760 PMC7419699

[B19] Le Cann S. Tudisco E. Turunen M. J. Patera A. Mokso R. Tägil M. (2019). Investigating the Mechanical Characteristics of Bone-Metal Implant Interface Using *In Situ* Synchrotron Tomographic Imaging. Front. Bioeng. Biotechnol. 6. 10.3389/fbioe.2018.00208 PMC6348316 30719433

[B20] Lievers W. B. Poljsak A. S. Waldman S. D. Pilkey A. K. (2010). Effects of Dehydration-Induced Structural and Material Changes on the Apparent Modulus of Cancellous Bone. Med. Eng. Phys. 32, 921–925. 10.1016/j.medengphy.2010.06.001 20638319

[B21] Lin C.-l. (2016). A Study of Human and Bovine Trabecular Bones Using Nanoindentation and Quantitative Backscattered Electron Imaging. Australia: The University of Queensland. 10.14264/uql.2016.164

[B22] Manda K. Xie S. Wallace R. J. Levrero-Florencio F. Pankaj P. (2016). Linear Viscoelasticity - Bone Volume Fraction Relationships of Bovine Trabecular Bone. Biomech. Model. Mechanobiol. 15, 1631–1640. 10.1007/s10237-016-0787-0 27090522 PMC5106511

[B23] Maquer G. Musy S. N. Wandel J. Gross T. Zysset P. K. (2015). Bone Volume Fraction and Fabric Anisotropy Are Better Determinants of Trabecular Bone Stiffness Than Other Morphological Variables. J. Bone Min. Res. 30, 1000–1008. 10.1002/jbmr.2437 25529534

[B24] Matsuura M. Eckstein F. Lochmüller E.-M. Zysset P. K. (2008). The Role of Fabric in the Quasi-Static Compressive Mechanical Properties of Human Trabecular Bone from Various Anatomical Locations. Biomech. Model. Mechanobiol. 7, 27–42. 10.1007/s10237-006-0073-7 17235622

[B25] Mays C. Bevitt J. Stilwell J. (2017). Pushing the Limits of Neutron Tomography in Palaeontology: Three-Dimensional Modelling of *In Situ* Resin within Fossil Plants. Palaeontol. Electron., 1–12. 10.26879/808

[B26] Morgan E. F. Bayraktar H. H. Keaveny T. M. (2003). Trabecular Bone Modulus-Density Relationships Depend on Anatomic Site. J. Biomechanics 36, 897–904. 10.1016/S0021-9290(03)00071-X 12757797

[B27] Nyman J. S. Gorochow L. E. Adam Horch R. Uppuganti S. Zein-Sabatto A. Manhard M. K. (2013). Partial Removal of Pore and Loosely Bound Water by Low-Energy Drying Decreases Cortical Bone Toughness in Young and Old Donors. J. Mech. Behav. Biomed. Mater. 22, 136–145. 10.1016/j.jmbbm.2012.08.013 23631897 PMC3655090

[B28] Nyman J. S. Roy A. Shen X. Acuna R. L. Tyler J. H. Wang X. (2006). The Influence of Water Removal on the Strength and Toughness of Cortical Bone. J. Biomechanics 39, 931–938. 10.1016/j.jbiomech.2005.01.012 PMC1941695 16488231

[B29] Oesch T. Weise F. Meinel D. Gollwitzer C. (2019). Quantitative *In-Situ* Analysis of Water Transport in Concrete Completed Using X-Ray Computed Tomography. Transp. Porous Med. 127, 371–389. 10.1007/s11242-018-1197-9

[B30] Perfect E. Cheng C.-L. Kang M. Bilheux H. Z. Lamanna J. M. Gragg M. J. (2014). Neutron Imaging of Hydrogen-Rich Fluids in Geomaterials and Engineered Porous Media: A Review. Earth-Science Rev. 129, 120–135. 10.1016/j.earscirev.2013.11.012

[B31] Raina D. B. Larsson D. Sezgin E. A. Isaksson H. Tägil M. Lidgren L. (2019). Biomodulation of an Implant for Enhanced Bone-Implant Anchorage. Acta Biomater. 96, 619–630. 10.1016/j.actbio.2019.07.009 31301423

[B32] Rho J.-Y. Kuhn-Spearing L. Zioupos P. (1998). Mechanical Properties and the Hierarchical Structure of Bone. Med. Eng. Phys. 20, 92–102. 10.1016/S1350-4533(98)00007-1 9679227

[B33] Roubin E. Andò E. Roux S. (2019). The Colours of Concrete as Seen by X-Rays and Neutrons. Cem. Concr. Compos. 104, 103336. 10.1016/j.cemconcomp.2019.103336

[B34] Ryd L. Albrektsson B. Carlsson L. Dansgard F. Herberts P. Lindstrand A. (1995). Roentgen Stereophotogrammetric Analysis as a Predictor of Mechanical Loosening of Knee Prostheses. J. Bone Jt. Surg. Br. 77-B, 377–383. 10.1302/0301-620x.77b3.7744919 7744919

[B35] Smith J. W. Walmsley R. (1959). Factors Affecting the Elasticity of Bone. J. Anat. 93, 503–523. 13832048 PMC1244544

[B36] Tengattini A. Lenoir N. Andò E. Giroud B. Atkins D. Beaucour J. (2020). NeXT-Grenoble, the Neutron and X-Ray Tomograph in Grenoble. Nucl. Instrum. Methods Phys. Res. Sect. A Accel. Spectrom. Detect. Assoc. Equip. 968, 163939. 10.1016/j.nima.2020.163939

[B37] Tengattini A. Lenoir N. Andò E. Viggiani G. (2021). Neutron Imaging for Geomechanics: A Review. Geomechanics Energy Environ. 27, 100206. 10.1016/j.gete.2020.100206

[B38] Törnquist E. Isaksson H. Turunen M. J. (2020). Mineralization of Cortical Bone during Maturation and Growth in Rabbits. J. Bone Min. Metab. 38, 289–298. 10.1007/s00774-019-01068-y 31807903

[B39] Törnquist E. Le Cann S. Tudisco E. Tengattini A. Andò E. Lenoir N. (2021). Dual Modality Neutron and X-Ray Tomography for Enhanced Image Analysis of the Bone-Metal Interface. Phys. Med. Biol. 66, 135016. 10.1088/1361-6560/ac02d4 34010812

[B40] Tötzke C. Kardjilov N. Lenoir N. Manke I. Oswald S. E. Tengattini A. (2019). What Comes NeXT? - High-Speed Neutron Tomography at ILL. Opt. Express 27, 28640. 10.1364/OE.27.028640 31684612

[B41] Urciuoli A. Zanolli C. Fortuny J. Almécija S. Schillinger B. Moyà‐Solà S. (2018). Neutron‐based Computed Microtomography: Pliobates Cataloniae and Barberapithecus Huerzeleri as a Test‐case Study. Am. J. Phys. Anthropol. 166, 987–993. 10.1002/ajpa.23467 29577230

[B42] van der Linden J. C. Weinans H. (2007). Effects of Microarchitecture on Bone Strength. Curr. Osteoporos. Rep. 5, 56–61. 10.1007/s11914-007-0003-3 17521506

[B43] Wang B. Chen R. Chen F. Dong J. Wu Z. Wang H. (2018). Effects of Moisture Content and Loading Profile on Changing Properties of Bone Micro-biomechanical Characteristics. Med. Sci. Monit. 24, 2252–2258. 10.12659/MSM.906910 29656299 PMC5917823

[B44] Zanolli C. Schillinger B. Beaudet A. Kullmer O. Macchiarelli R. Mancini L. (2017). Exploring Hominin and Non-hominin Primate Dental Fossil Remains with Neutron Microtomography. Phys. Procedia 88, 109–115. 10.1016/j.phpro.2017.06.014

[B45] Zanolli C. Schillinger B. Kullmer O. Schrenk F. Kelley J. Rössner G. E. (2020). When X-Rays Do Not Work. Characterizing the Internal Structure of Fossil Hominid Dentognathic Remains Using High-Resolution Neutron Microtomographic Imaging. Front. Ecol. Evol. 8. 10.3389/fevo.2020.00042

