# Supplementary material for: The Hydration State of Bone Tissue Affects Contrast in Neutron Tomographic Images
Source: Front Bioeng Biotechnol. 2022 Jun 17;10:911866. doi: 10.3389/fbioe.2022.911866 (PMC9247154; doi:10.3389/fbioe.2022.911866)
Supplement: Supplementary file 1 [file DataSheet1.pdf]

doi: 10.3389/fbioe.2022.911866

Supplementary Material

The hydration state of bone tissue affects contrast in neutron tomographic images

1 Tomographic images of bovine trabecular bone plugs

Bovine trabecular bone plugs were imaged with neutron and X-ray tomography at the NeXT-Grenoble beamline at ILL, France, and with X-ray tomography at the 4D Imaging Lab in Lund, Sweden. At NeXT-Grenoble, the plugs were imaged at different hydration states (soaked in D_2O (control), dry, rehydrated) whilst the plugs imaged at the 4D Imaging Lab were imaged before being subjected to drying and rehydration. Supplementary Figure 1 shows a comparison between tomographic images of plugs from the control group. The isotropic voxel size in the tomographic images were 15.6 µm for the neutron images (Supplementary Figure 1A), 24 µm for the X-ray images acquired at NeXT-Grenoble (Supplementary Figure 1B), and 13.5 µm for the X-ray images acquired at the 4D imaging Lab (Supplementary Figure 1C).

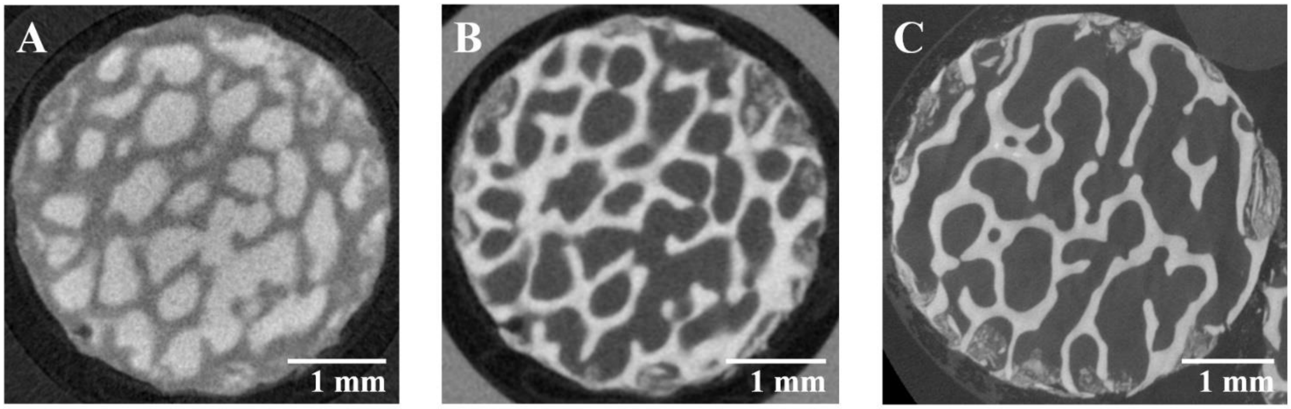

Supplementary Figure 1. Comparison between tomographic images of bovine trabecular bone plugs. A) Neutron image of a representative specimen from the control (soaked in D_2O) group. B) X-ray image from NeXT-Grenoble of the same specimen as in A. C) X-ray image of a representative specimen imaged at the 4D Imaging Lab (note that this is a different specimen than that shown in A and B). The gray value range displayed in A and B is [14500,55000] and [45000,65535] in C (all images are in 16-bits). Notice how the inter-trabecular marrow is clearly visualized in the neutron images (high intensities) whilst it appears as the same intensities as void/background (low intensities) in the X-ray images.

2 Statistical analysis of effects of hydration state on mechanical properties

A linear mixed-effects model was used to assess effects of hydration state on mechanical properties. To correct for heteroskedasticity in the residuals, the response variable (mechanical property) was log-transformed. The resulting scatter plots for the residuals as a function of the fitted response values are shown in Supplementary Figure 2A. Histograms of the residuals, displaying normal distributions, are shown in Supplementary Figure 2B. Supplementary Figure 2C shows how the resulting fits correspond well with the response.

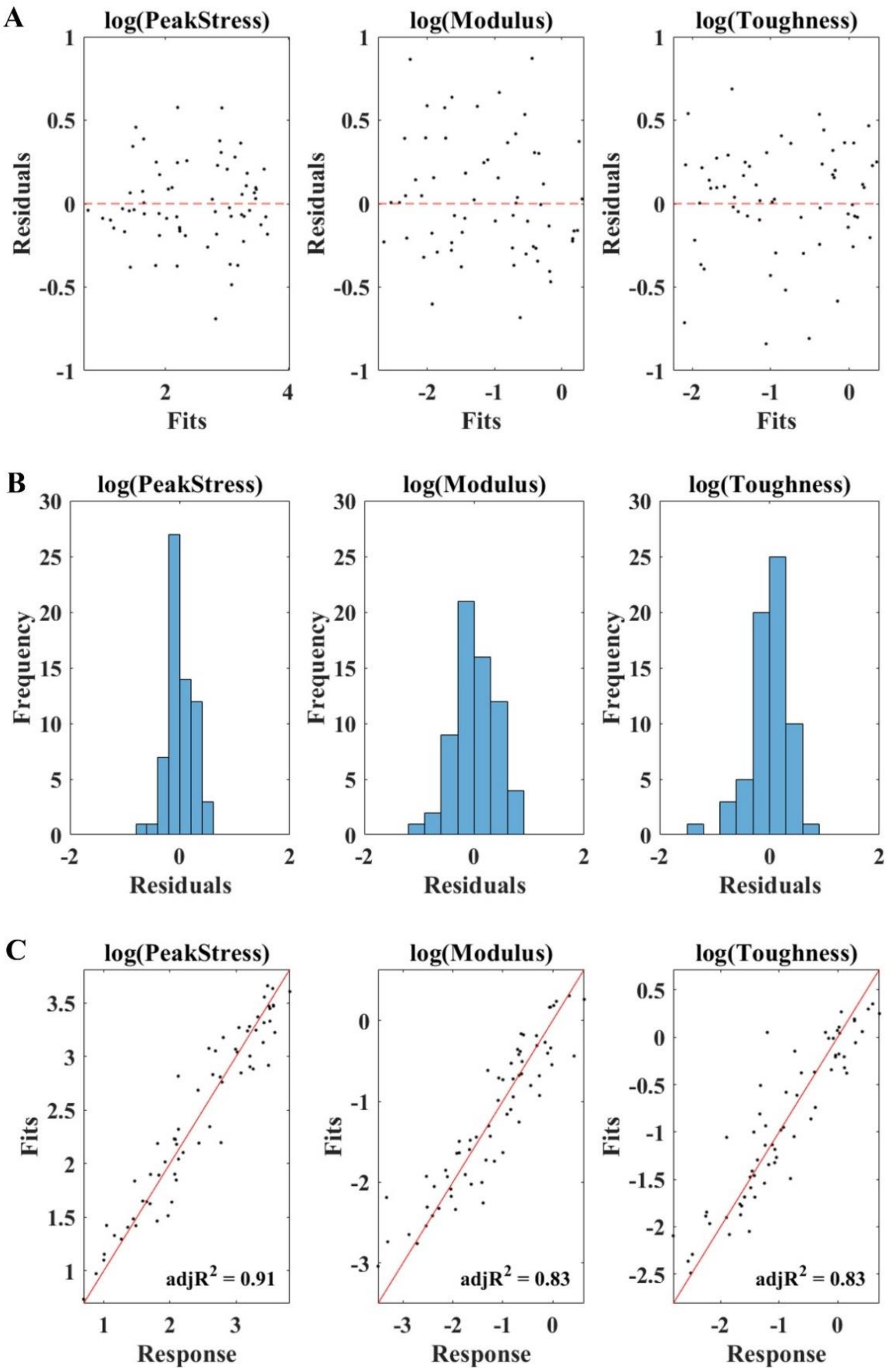

Supplementary Figure 2. Residual analysis. A) Homoskedasticity in the residuals after log-transformation of the response variables (mechanical properties). B) Histograms of the residuals, showing normal distributions. C) Comparison of the fitted values to the response values. The distribution follows the 45° line in red well, which, together with the high values of the adjusted coefficient of determination (adjR^2), indicate high goodness-of-fit.
